# Editorial: Subclinical thyroid disease: present knowledge and future direction

**DOI:** 10.3389/fendo.2022.980585

**Published:** 2022-07-21

**Authors:** Jose De Jesus Garduno Garcia, Alberto O. Chavez, Daniel Elías-López, Iván Pérez-Díaz

**Affiliations:** ^1^ Medicine, Universidad Autónoma del Estado de México, Toluca, Mexico; ^2^ Internal Medicine, Instituto Mexicano del Seguro Social (IMSS), Toluca, Mexico; ^3^ Texas Diabetes Institute - University Health, UT Health San Antonio, San Antonio, TX, United States; ^4^ Internal Medicine, Instituto Nacional de Ciencias Médicas y Nutrición Salvador Zubirán (INCMNSZ), Mexico City, Mexico; ^5^ Department of Medicine, Tecnologico de Monterrey School of Medicine and Health Science, Mexico city, Mexico

**Keywords:** thyroid, subclinical thyroid disease, thyroid and pregnancy, thyroid and cardiovascular disease, mild thyroid dysfunction

For centuries, a wide spectrum of thyroid dysfunction has been described in the literature across different civilizations. Interestingly, our current understanding about the structural, functional, and pathophysiologic aspects underlying thyroid disease has only fundamentally changed in recent decades as new technologies are developed, in addition to the increasing amount of data from clinical trials. These discoveries have led to a paradigm change in the management of thyroid disease (1).

Mild to moderate thyroid dysfunction is commonly found during routine testing in clinical practice, affecting between 3-8% of the general population. There are numerous pathophysiological mechanisms described in the literature to establish a clear relationship between thyroid dysfunction and the development of various pathologies such as obesity, depression, cardiovascular disease, and dyslipidemia. Untreated subclinical hypothyroidism (SCH), for instance, has been linked with a number of deleterious outcomes, although its weight as a disease modifying factor is still subject of controversy in some specific scenarios (2).

Altogether, the emergence of subclinical thyroid disease as a significant contributor to other disease states has opened multiple lines of research and the expanding literature on this topic is a welcome addition to the existing body of knowledge available for the clinician, particularly in times when misinformation abounds among the general population regarding the definition of true thyroid illness (3). In the current Research Topic, different groups of investigators explore various interesting questions and generate novel knowledge regarding this wide group of conditions.

One of the major challenges in subclinical thyroid disease is the need to establish a TSH cut-off point to define SCH. In this issue, Zheng et al. describe the association between thyroid autoantibodies distribution in relation to different TSH cut-off values in a large cohort of 145,015 patients. Another area of subject of intense research is the effect that SCH has in pregnancy related outcomes (4, 5). Meng et al. propose a nomogram that considers the effect of TSH and TPO antibodies and the interaction with other factors, such as mother age, that could predict an increased risk of preterm delivery. In the same pregnancy related topic, Zhou et al. describe the possible relationship between Free T4 levels in the first trimester and the risk of preterm delivery. Moreover, the benefit of treating antibody positive non-hypothyroid pregnant women with thyroid hormone replacement is an area of investigation that has not been studied extensively. A clinical trial by Li et al. demonstrated no difference in the incidence of hypertensive disorders during pregnancy, but a reduction in miscarriage occurrence was observed. In these reports several other materno-fetal outcomes are also investigated. Furthermore, a meta-analysis by Han et al. explores the effect of SCH in pregnant and non-pregnant women in the overall risk of developing hypertensive disorders.

Although the neurocognitive effects in the offspring of women with uncontrolled established hypothyroidism are well known, the effect of mild thyroid dysfunction in pregnant women without TPO antibodies is less understood. In the article by Wang et al. the presence of maternal mild thyroid dysfunction was associated to an impaired neurocognitive function, manifested as lower receptive communication score at one year of age when compared with children from women with normal thyroid function, at one year of age.

Cardiovascular disease remains the number one cause of death worldwide (6, 7). Thyroid dysfunction, specifically overt hypothyroidism is linked to a worse cardiovascular risk profile through several mechanisms, such as impaired lipid metabolism causing hypercholesterolemia (8). The effect of mild thyroid dysfunction, however, as a cardiovascular risk enhancer is less clear (9). In their paper, Li et al. compared a group of patients who presented with confirmed acute ST-segment elevation myocardial infarction, based on the presence of subclinical thyroid disease (both hyper and hypothyroid) and compared post-event outcomes with those individuals with normal thyroid function. After adjustments for other risk factors, there was an increment in hospital cardiovascular deaths in the group with subclinical hyperthyroidism. In the other hand, Meng et al. investigated the incidence of atrial fibrillation development in a group of patients with hypertrophic obstructive cardiomyopathy and low TSH. Given the complex and multifactorial pathogenesis of cardiovascular disease, it is frequently difficult to isolate the dominant factor leading to an excessive cardiac risk. An impaired coagulation is one of such risk factors. It is known that thyroid function also effects the coagulation and fibrinolytic system, and thyroid dysfunction is associated to a hypercoagulation state. The detail of this intricate relationship remains to be fully elucidated, but on systematic review by Xu et al. they analyzed data on 1325 patients from 12 observational studies and describe the pattern of disrupted homeostasis between hemostatic biomarkers in subjects with abnormal thyroid function.

Subclinical thyroid disease represents an important challenge in clinical medicine. Mounting evidence strongly indicates a pressing need for earlier and better screening in vulnerable populations likely to experience adverse outcomes, such as pregnant women and individuals with a high cardiovascular risk. Research is underway aiming to elucidate the specific mechanisms responsible for these interactions and to identify those patients more likely to benefit from early thyroid replacement and the optimal timing for intervention needed to reduce such outcomes.

## Author contributions

All authors listed have made a substantial, direct, and intellectual contribution to the work and approved it for publication.

## Conflict of interest

The authors declare that the research was conducted in the absence of any commercial or financial relationships that could be interpreted as a potential conflict of interest.

## Publisher’s note

All claims expressed in this article are solely those of the authors and do not necessarily represent those of their affiliated organizations, or those of the publisher, the editors and the reviewers. Any product that may be evaluated in this article, or claim that may be made by its manufacturer, is not guaranteed or endorsed by the publisher.
